# Defining clinically meaningful values in the oxford hip score and factors associated with their achievement following aseptic revision total hip arthroplasty

**DOI:** 10.1007/s00264-026-06808-0

**Published:** 2026-04-30

**Authors:** Thomas R. Williamson, Paul Gaston, Gavin J. MacPherson, James T. Paton, Philip M. S. Simpson, Andrew D. Duckworth, Chloe E. H. Scott, Nick D. Clement

**Affiliations:** 1 Edinburgh Orthopaedics, Royal Infirmary of Edinburgh, Edinburgh, United Kingdom; 2 School of Population Health Sciences, Usher Institute, University of Edinburgh, Edinburgh, United Kingdom; 3 Department of Mechanical Engineering, Imperial College London, London, United Kingdom

**Keywords:** Revision, Hip arthroplasty, Clinically meaningful values, Patient reported outcome measures, PASS, MIC

## Abstract

**Purpose:**

To define the ‘patient acceptable symptom state’ (PASS) and ‘minimum important change’ (MIC) for the Oxford Hip Score (OHS) following aseptic revision total hip arthroplasty (rTHA), and identify factors associated with their achievement.

**Methods:**

A prospective cohort of 135 patients (138 hips) undergoing aseptic rTHA at a single centre were followed up at one and two years postoperatively. Demographics, health-related quality of life (HRQoL; EQ-5D) and OHS were recorded at each timepoint. Anchor techniques were used to define the MIC and PASS. Regression models identified factors associated with PASS and MIC achievement.

**Results:**

The OHS PASS was 31.5 and 33.5 at one and two years postoperatively, respectively. The MIC was 8.5 at both timepoints. A greater preoperative EQ-5D was independently associated with PASS achievement at both timepoints. One-year MIC achievement was independently associated with lower BMI (*p* = 0.042) and lower preoperative OHS (*p* = 0.007), whilst lower preoperative OHS (*p* = 0.016) alone was independently associated with two year MIC achievement (*p* = 0.016). Lower preoperative EQ-5D and ASA grade 3 were associated with failure to achieve either PASS or MIC at one year (*p* = 0.030) and two years (*p* = 0.013) postoperatively, respectively.

**Conclusion:**

The PASS and MIC thresholds for the OHS following aseptic rTHA contextualise the score and can inform study design. Greater preoperative HRQoL was independently associated with PASS achievement, whilst worse preoperative function was independently associated with MIC achievement. These thresholds should be considered in conjunction when assessing outcomes following aseptic rTHA.

## Introduction

Patient-reported outcome measures (PROMs) are widely used tools to evaluate outcomes following total hip arthroplasty (THA) [1]. Outcomes following revision THA (rTHA) are typically inferior to those reported after primary THA [2]. However, PROMs initially employed to assess primary THA have been validated for this population, such as the Oxford Hip score (OHS) [3]. Assessment of patients’ outcomes following rTHA is central to both informing clinical practice and evaluating different implants and surgical techniques.

Clinically meaningful values (CMVs), such as the ‘patient acceptable symptom state’ (PASS) and the ‘minimum important change’ (MIC) are used to contextualise PROMs [4]. The PASS refers to a threshold on a PROM scale beyond which a patient can be expected to have achieved a satisfactory outcome [5]. Similarly, the MIC refers to the smallest change in PROM score over time above which a patient feels they have had a meaningful change [6]. The PASS threshold for the OHS following primary THA has been reported to be 40 and 39 at one and two years postoperatively respectively, but has not been defined following rTHA [7]. Given the inferior outcomes observed following rTHA, CMVs defined for primary THA are unlikely to be generalisable to this population [2]. Indeed, the MIC for the OHS at six months following rTHA is lower than that reported for primary THA [8]. MIC values following primary THA have been shown to increase up to 12 months postoperatively with a plateau thereafter, mirroring the changes observed in OHS postoperatively [9, 10]. It remains unclear what the PASS and MIC thresholds are for the OHS following aseptic rTHA at one and two years postoperatively, and whether a similar plateau in threshold is observed.

Understanding the factors associated with achieving the PASS and MIC thresholds following rTHA could enhance preoperative counselling. There is limited evidence identifying factors associated with the achievement of CMV thresholds following rTHA, but greater baseline function and surgical approach are associated with postoperative functional outcomes [11]. The heterogeneity of surgical indications for rTHA should also be considered when assessing outcomes postoperatively, as aseptic rTHA is associated with superior functional outcomes than rTHA performed for infection [12].

This study aimed to define the one and two year PASS and MIC for the OHS following aseptic rTHA using the same implant, and to identify factors associated with achieving these CMVs.

## Methods

Local ethical approvals were sought before study commencement (*BLINDED FOR REVIEW*) and the study was conducted in accordance with the Declaration of Helsinki. The study cohort formed part of the *[BLINDED FOR REVIEW]* project, prospectively followed up at one and two years postoperatively. A consecutive series of 135 patients (138 hips) undergoing aseptic rTHA using a Tritanium Trident II® acetabular shell (Stryker, Mahwah, NJ) through a posterior approach were prospectively enrolled between January 2020 and 2023 from a single centre. The surgical technique has been previously described [13]. Patients were primarily female (n = 78, 56.5%), with a mean age of 71 (SD 13.5) years. Demographic variables, comorbidities and surgical indications are presented in Table 1.

**Table 1:** Patient demographics and implant characteristics

	*n* = 138
**Age (years, SD)**	71 (13.5)
**Sex (F, %)**	78 (56.5)
**BMI (Kg/m**^**2**^**, SD)**	28.8 (5.4)
**SIMD (IQR)**	7 (4–9)
**ASA grade (n, %)**	
* I*	12 (8.7)
* II*	88 (63.8)
* III*	38 (27.5)
**Charlson Comorbidity Index (IQR)**	3 (2–4)
**Paprosky Classification (n, %)**	
* 1*	49 (35.5)
* 2A*	35 (25.4)
* 2B*	24 (17.4)
* 2C*	18 (13)
* 3A*	7 (5.1)
* 3B*	5 (3.6)
**Revision Indication (n, %)**	
* Aseptic loosening*	113 (81.9)
* Instability*	16 (11.6)
* Osteolysis*	2 (1.4)
* Pain*	3 (2.2)
* Failed femoral component*	2 (1.4)
* Periprosthetic fracture*	1 (0.7)
* Component wear*	1 (0.7)

*F* Female, *BMI* Body mass index, *SIMD* Scottish Index of Multiple Deprivation, *ASA* American Society of Anesthesiologists

### Data collection

Patients’ demographic and comorbidity data, including age, sex, body mass index (BMI), and socioeconomic deprivation index decile (SIMD, Scottish Index of Multiple Deprivation; 1 representing the most deprived) were recorded preoperatively. Acetabular bone loss was quantified using the Paprosky classification [14]. Preoperative PROMs were recorded using questionnaires two to six weeks in advance of their operation. Patients were followed-up at one and two years postoperatively using similar questionnaires. Frequency of re-revision arthroplasty, complications and mortality were recorded from patients’ electronic medical records.

PROMs included the OHS, EQ-5D-3L, and EQ VAS. The OHS is a 12-item score assessing joint-specific function, scored from 0 to 48, with 0 representing the worst outcome [15]. The EQ-5D assesses health-related quality of life (HRQoL) across five domains (−5D). This ranges from −0.594 to 1.0, with 1 representing the best health, and scores below 0 representing a health state ‘worse than death’ [16]. The EQ VAS assesses HRQoL using a visual analogue scale (VAS), with scores ranging from 0 to 100 (best health) [17]. Satisfaction was assessed using the question ‘*How satisfied are you with your operated hip*?’, with responses recorded using a 5-item Likert scale including ‘Very Satisfied’, ‘Satisfied’, ‘Neutral’, ‘Dissatisfied’, and ‘Very Dissatisfied’. Responses were dichotomised, with the answers ‘Very Satisfied’ and ‘Satisfied’ defined as satisfied, and ‘Neutral’, ‘Dissatisfied’, or ‘Very Dissatisfied’ defined as not satisfied.

### Statistical analysis

Statistical analyses were performed using Statistical Package for Social Sciences version 29.0.1.0 (IBM Inc., Armonk, NY, USA). Normality was assessed using Shapiro–Wilk testing, with parametric data reported using mean and standard deviation (SD), and nonparametric data reported using median and interquartile range (IQR). CMV thresholds were calculated using receiver operator curve analysis and Youden’s index, with satisfaction as anchor. Sensitivity, specificity, and the Area Under the Curve (AUC) were reported. Outcomes were compared between groups using independent T-tests and Chi-squared tests, or Mann–Whitney U-tests for nonparametric analyses. Logistic regression models predicted CMV achievement, with variables associated with CMV achievement in unadjusted analysis eligible for inclusion. Final models were defined using the backward Likelihood Ratio method [18]. Model fit was assessed using Nagelkerke pseudo-R^2^. p < 0.05 was considered significant for all analyses.

## Results

At two year follow-up, survivorship data were complete for 136 hips (98.6%). Six patients were deceased (3.8%). Three hips had undergone re-revision (2.2%): one for infection, one for periprosthetic acetabular fracture, and one for aseptic loosening. Early closed reduction for dislocation was performed for two hips (1.4%) within three months postoperatively.

PROMs were complete for 81 patients at one year postoperatively and 71 patients at two years postoperatively. The OHS increased from 24.3 (SD 11.5) preoperatively to 37.9 (SD 10.7) at one year (p < 0.001) and 36.4 (SD 11.6) at two years postoperatively (p = 0.002). EQ-5D index increased from 0.523 (SD 0.462) preoperatively to 0.875 (SD 0.173) at one year (p < 0.001) and 0.729 (SD 0.240) at two years postoperatively (p < 0.001). 61 patients (83.6%) were satisfied at one year follow-up, and 56 (78.9%) were satisfied at two year follow-up.

### Clinically meaningful values

**Fig. 1:**
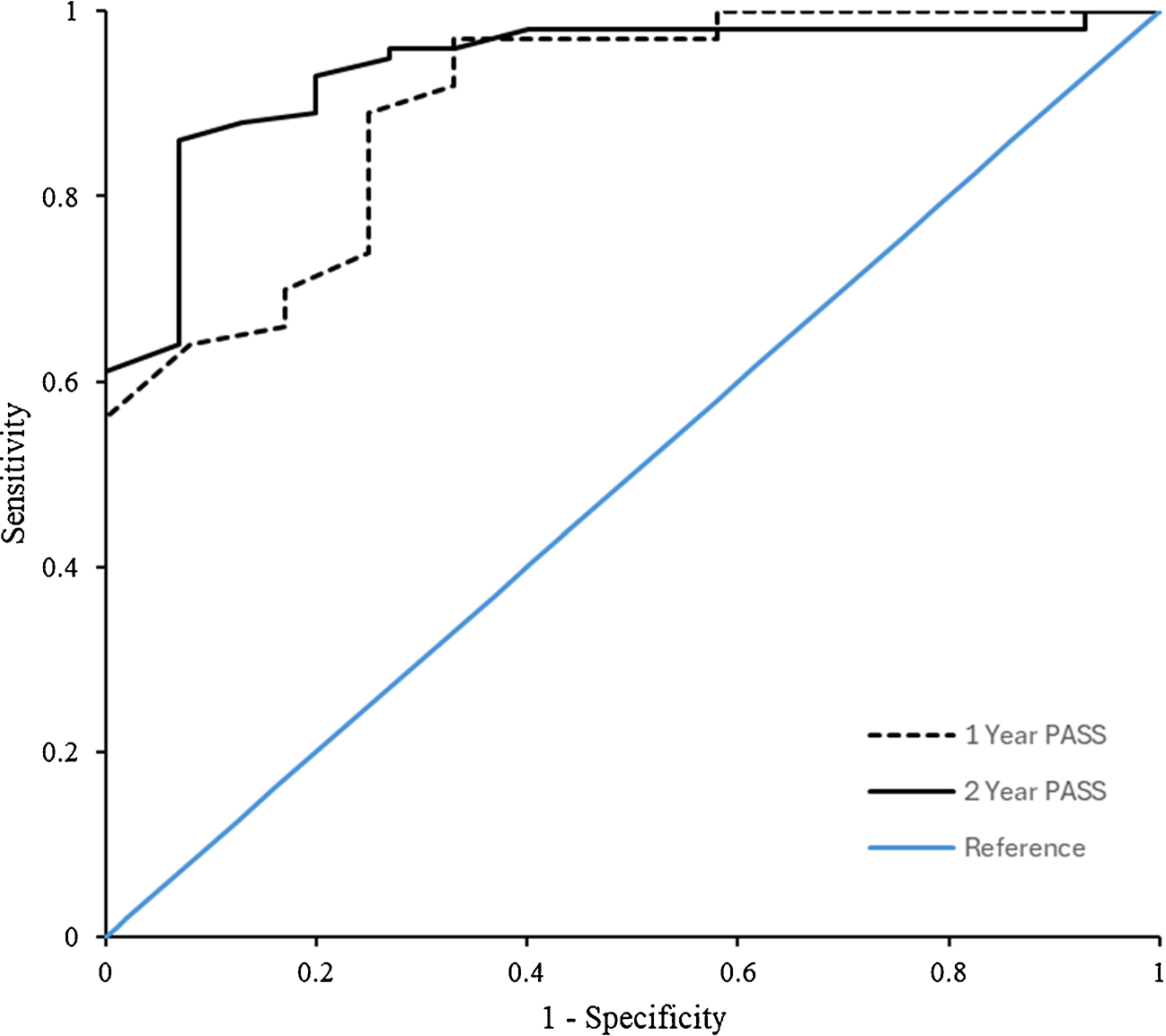
Receiver operator curves defining the 1- and 2-year OHS PASS

**Fig. 2:**
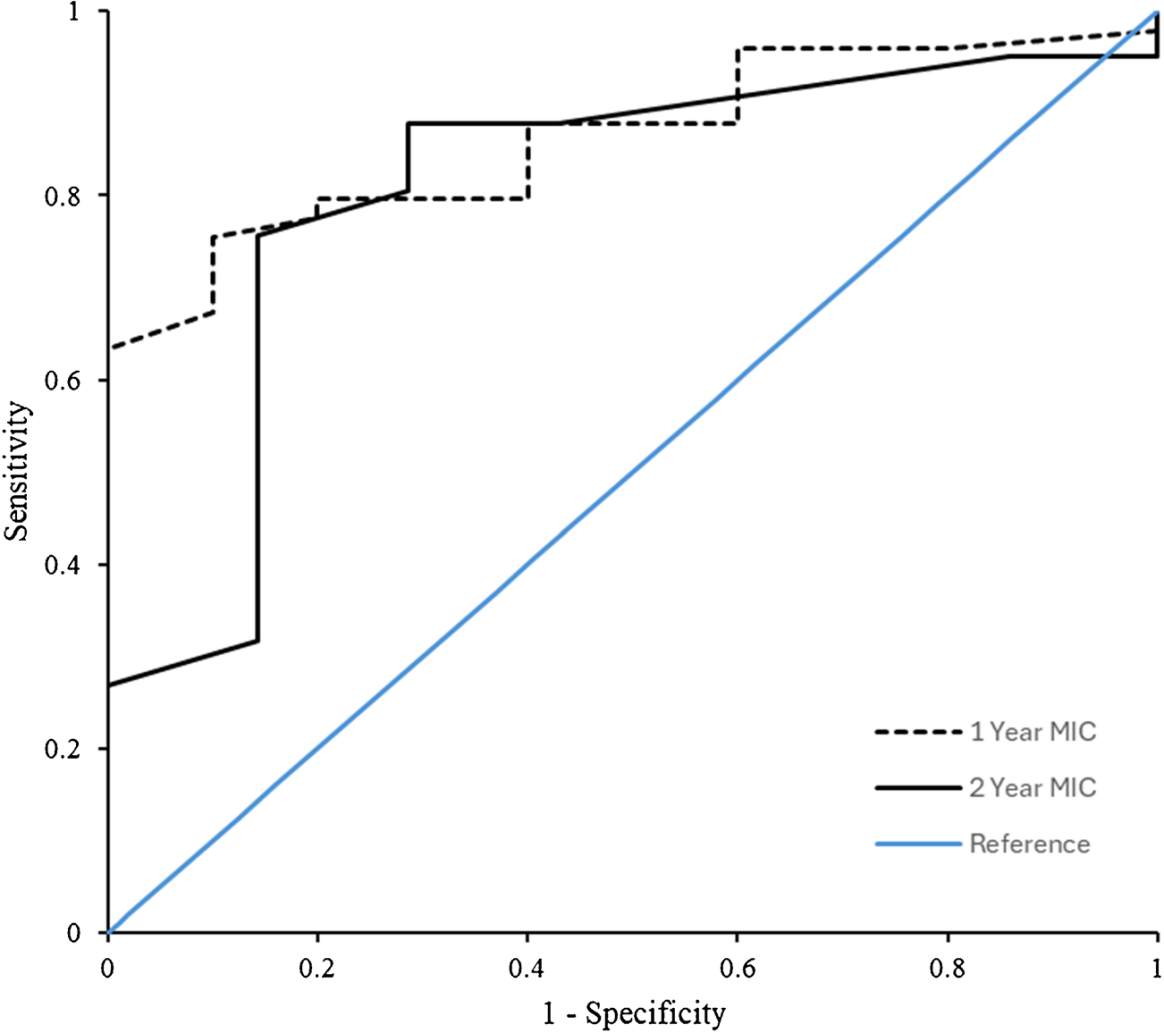
Receiver operator curves defining the 1- and 2-year OHS MIC

The PASS threshold for the OHS at 1-year postoperatively was 31.5 (AUC 0.898, 95% CI: 0.806 to 0.989; sensitivity 88.5%, specificity 75%), and at two years postoperatively it was 33.5 (AUC 0.940, 95% CI: 0.880 to 1.0; sensitivity 85.7%, specificity 93.3%) (Fig. 1). The MIC threshold for the OHS was 8.5 at both at one year (AUC 0.862, 95% CI: 0.766 to 0.959; sensitivity 75.5%, specificity 90%) and two years postoperatively (AUC 0.807, 95% CI: 0.628 to 0.985; sensitivity 75.6%, specificity 85.7%) (Fig. 2).

### Factors associated with PASS achievement

The PASS threshold was achieved by 62 patients (76.5%) at one year, and 49 patients (69%) at two years postoperatively. Of the patients failing to achieve PASS, seven (43.8%) were satisfied at one year, and eight (36.4%) at two year follow-up. Patients achieving PASS at one year postoperatively had a significantly greater preoperative EQ-5D index and OHS. Patients achieving PASS at two year follow-up had a significantly greater preoperative EQ-5D index and OHS, greater SIMD, and were more frequently male (Table 2).

**Table 2:** Preoperative PROMs and patient characteristics associated with OHS PASS achievement

	1 Year OHS PASS			2 Year OHS PASS		
	Achieved (*n* = 62)	Not Achieved (*n* = 19)	*p*-value	Achieved (*n* = 49)	Not Achieved (*n* = 22)	*p*-value
**Age (years, SD)**	71.4 (11.4)	71.4 (13.8)	*0.923*	71.6 (11.7)	70.7 (12.6)	*0.597*
**Sex (F, %)**	31 (50)	13 (68.4)	*0.158*	23 (46.9)	18 (81.8)	*0.006*
**BMI (n, %)**			*0.079*			*0.304*
** < 30 kg/m**^**2**^	44 (72.1)	9 (50)		35 (71.4)	13 (59.1)	
** ≥ 30 kg/m**^**2**^	17 (27.9)	9 (50)		14 (28.6)	9 (40.9)	
**SIMD Decile (IQR)**	7.5 (5–10)	5 (5–7.5)	*0.365*	7.5 (5.5–10)	5 (5–8)	*0.004*
**ASA Grade (n, %)**			*0.700*			*0.298*
** I**	6 (9.7)	1 (5.3)		5 (10.2)	1 (4.5)	
** II**	42 (67.7)	12 (63.2)		35 (71.4)	13 (59.1)	
** III**	14 (22.6)	6 (31.6)		9 (18.4)	8 (36.4)	
**EQ-5D Index (median, IQR)**	0.796 (0.159–1.0)	−0.248 (−0.426 – 0.119)	< *0.001*	0.796 (0.212-1.0)	0.055 (-0.337 - 0.139)	< *0.001*
**EQ VAS (median, IQR)**	80 (66—90)	65 (52–78.5)	*0.054*	80 (65.5–90)	73.5 (54–80)	*0.279*
**OHS (mean, SD)**	26.6 (9.7)	14.4 (10.5)	*0.010*	26.7 (10.0)	16.4 (10.3)	*0.004*

*F* Female, *BMI* Body mass index, *SIMD* Scottish Index of Multiple Deprivation, *ASA* American Society of Anaesthesiologists, *EQ-5D* EuroQol-5 dimension index, *OHS* Oxford hip score, *PASS* Patient acceptable symptom state

The regression models predicting one and two year PASS achievement were able to predict group membership (*p* < 0.001) (Table 3). Preoperative EQ-5D index scores of ≥ 0.165 (AUC 0.820, 95% CI: 0.693 to 0.947, *p* < 0.001), and ≥ 0.178 (AUC 0.848, 95% CI: 0.708 to 0.987, *p* < 0.001) were excellent predictors of one and two year OHS PASS achievement respectively.

**Table 3:** Logistic regression model predicting 1 and 2-year PASS achievement

		B	SE	Wald	OR (95% CI)	*p-value*
**1-year**	**Constant**	0.506	0.405	1.564	1.659	0.211
	EQ-5D^*^	0.257	0.082	9.917	1.293 (1.102 to 1.518)	0.002
*Nagelkerke R*^*2*^ = *0.303; 86.9% of cases correctly classified*						
**2-year**	**Constant**	0.183	0.436	1.075	1.2	0.676
	EQ-5D^*^	0.312	0.096	10.566	1.367 (1.132 to 1.650)	0.001
*Nagelkerke R*^*2*^ = *0.414; 82% of cases correctly classified*						

*OR* Odds Ratio, *SE* Standard error, *EQ-5D* EuroQol 5-dimension index

^*^EQ-5D scaled by a factor of 10 to aid interpretation: An increase in EQ-5D index of 0.1 equates to a 1.293 times increase in odds of OHS PASS achievement

### Factors associated with MIC achievement

The OHS MIC was achieved by 42 patients (66.7%) at one year, and 32 patients (66.7%) at two years postoperatively. Of the patients failing to achieve MIC, 12 (57.1%) were satisfied at one year postoperatively, and ten (62.5%) at two years. Patients achieving MIC at both one and two years postoperatively had a significantly lower BMI and preoperative OHS (Table 4).

**Table 4:** Preoperative PROMs and patient characteristics associated with OHS MIC achievement

	1 Year OHS MIC			2 Year OHS MIC		
	Achieved (*n* = 42)	Not Achieved (n = 21)	*p*-value	Achieved (*n* = 32)	Not Achieved (*n* = 16)	*p*-value
**Age (years, SD)**	69 (16.2)	72.8 (13.6)	*0.362*	71.5 (11.8)	70.3 (12.5)	*0.739*
**Sex (F, %)**	24 (57.1)	12 (57.1)	*1.000*	18 (56.3)	10 (62.5)	*0.679*
**BMI (n, %)**			*0.044*			*0.279*
** < 30 kg/m**^**2**^	30 (71.4)	9 (45)		23 (71.9)	9 (56.3)	
** ≥ 30 kg/m**^**2**^	12 (28.6)	11 (55)		9 (28.1)	7 (43.4)	
**SIMD Decile (IQR)**	7 (4–9)	7 (5–8)	*0.831*	7 (4–9.5)	6 (5.5–8)	*0.749*
**ASA Grade (n, %)**			*1.000*			*0.649*
** I**	4 (9.5)	2 (9.5)		3 (9.4)	2 (12.5)	
** II**	29 (69)	14 (66.7)		22 (68.7)	9 (56.3)	
** III**	9 (21.5)	5 (23.8)		7 (21.9)	5 (31.3)	
**EQ-5D Index (median, IQR)**	0.691 (0.088–0.85)	0.390 (−0.08 – 1.0)	*0.242*	0.426 (0.072–0.796)	0.942 (0.119–1.0)	*0.680*
**EQ-5D VAS (median, IQR)**	77.5 (60–90)	80 (69–80)	*0.708*	70.5 (52–90)	80 (68–85)	*0.473*
**OHS (mean, SD)**	22.7 (7.4)	31.6 (14.3)	*0.013*	21.4 (7)	29.9 (14.7)	*0.041*

*F* Female, *BMI* Body mass index, *SIMD* Scottish Index of Multiple Deprivation, *ASA* American Society of Anesthesiologists, *EQ-5D* EuroQol-5 dimension index, *OHS* Oxford hip score, *MIC* Minimal important change

The regression models predicting one and two year MIC achievement were able to predict group membership (*p* < 0.001) (Table 5). Preoperative OHS of ≤ 30.5 (AUC 0.736, 95% CI: 0.572 to 0.901, *p* = 0.005), and ≤ 29.5 (AUC 0.696, 95% CI 0.503 to 0.890, *p* = 0.047) were acceptable and poor predictors of two year MIC achievement respectively.

**Table 5:** Logistic regression model predicting 1 and 2-year MIC achievement

		B	SE	Wald	OR (95% CI)	*p*-value
**1-year**	**Constant**	2.912	0.929	9.819	18.388	0.002
	**BMI** ≥ **30 kg/m**^**2**^	−1.254	0.617	4.130	0.285 (0.085 to 0.956)	0.042
	**OHS**	−0.085	0.032	7.226	0.919 (0.864 to 0.977)	0.007
*Nagelkerke R*^*2*^ = *0.257; 79% of cases correctly classified*						
**2-year**	**Constant**	2.759	0.946	8.507	15.777	0.004
	**OHS**	−0.081	0.034	5.822	0.922 (0.864 to 0.985)	0.016
*Nagelkerke R*^*2*^ = *0.187; 83.3% of cases correctly classified*						

*OR* Odds Ratio, *SE* Standard error, *BMI* Body mass index, *OHS* Oxford hip score

### Factors associated with failure to achieve either MIC or PASS

At one year follow-up, 54 hips (94.7%) achieving PASS, and 37 (97.4%) achieving MIC were satisfied. Nine (14.3%) failed to achieve either the PASS or MIC, of which two (22.2%) were satisfied (*p* < 0.001). At two year follow-up, eight hips (16.7%) failed to achieve either the PASS or MIC, of which two (25%) were satisfied (*p* < 0.001). Factors associated with failure to achieve either threshold in unadjusted analyses at one and two years postoperatively are presented in Table 6.

**Table 6:** Preoperative PROMs and patient characteristics associated with failure to achieve either OHS MIC or PASS

	1 Year OHS PASS or MIC			2 Year OHS PASS or MIC		
	Achieved (*n* = 54)	Not Achieved (*n* = 9)	*p*-value	Achieved (*n* = 40)	Not Achieved (*n* = 8)	*p*-value
**Age (years, SD)**	70.6 (14.7)	68.3 (20.1)	*0.677*	70.7 (12)	72.8 (11.8)	*0.653*
**Sex (F, %)**	30 (55.6)	6 (66.7)	*0.533*	22 (55)	6 (75)	*0.440*
**BMI (n, %)**			*0.066*			*0.413*
** < 30 kg/m**^**2**^	36 (67.9)	3 (33.3)		28 (70)	4 (50)	
** ≥ 30 kg/m**^**2**^	17 (32.1)	6 (66.7)		12 (30)	4 (50)	
**SIMD Decile (IQR)**	7 (4–8.5)	6 (5–9)	*0.670*	7 (4.5–9.5)	5.5 (5–7)	*0.333*
**ASA Grade (n, %)**			*0.166*			*0.016*
** I**	5 (9.3)	1 (11.1)		4 (10)	1 (12.5)	
** II**	39 (72.2)	4 (44.4)		29 (72.5)	2 (25)	
** III**	10 (18.5)	4 (44.4)		7 (17.5)	5 (62.5)	
**EQ-5D Index (median, IQR)**	0.796 (0.130 to 1.0)	0.088 (−0.248 to 0.139)	*0.020*	0.727 (0.095 to 1.0)	0.119 (−0.248 to 0.390)	*0.061*
**EQ-5D VAS (median, IQR)**	80 (65.5–90)	70 (54–80)	*0.179*	80 (60–90)	73.5 (45–80)	*0.398*
**OHS (mean, SD)**	26.7 (10.5)	19.4 (12.6)	*0.067*	25.5 (10.6)	18.1 (10.8)	*0.081*

*F* Female, *BMI* Body mass index, *SIMD* Scottish Index of Multiple Deprivation, *ASA* American Society of Anesthesiologists, *EQ-5D* EuroQol-5 dimension index, *OHS* Oxford hip score, *PASS* Patient acceptable symptom state, *MIC* Minimal important change

The regression models predicting failure to achieve either MIC or PASS at one and two year assessment were significantly able to predict group membership (*p* = 0.022) (Table 7). A preoperative EQ-5D of < 0.165 was an acceptable predictor of failure to achieve either CMV at one year postoperatively (AUC 0.734, 95% CI: 0.570 to 0.898, *p* = 0.005). ASA grade 3 was independently associated with failure to achieve either CMV at two years postoperatively.

**Table 7:** Logistic regression model predicting failure to achieve 1 year MIC or PASS

		B	SE	Wald	OR (95% CI)	*p*-value
**1-year**	**Constant**	−1.118	0.426	6.894	0.327	0.009
	EQ-5D^*^	−0.168	0.078	4.692	0.845 (0.726 to 0.984)	0.030
*Nagelkerke R*^*2*^ = *0.142; 85.2% of cases correctly classified*						
**2-year**	**Constant**	−2.674	0.731	13.379	0.069	< 0.001
	**ASA**^**+**^			6.241		0.044
	**I**	1.288	1.336	0.929	3.625 (0.264 to 49.703)	0.335
	**II**	Reference				
	**III**	2.338	0.937	6.229	10.357 (1.652 to 64.943)	0.013
*Nagelkerke R*^*2*^ = *0.232; 83.3% of cases correctly classified*						

*OR* Odds Ratio, *SE* Standard error, *EQ-5D* EuroQol 5-dimension index

^*^EQ-5D scaled by a factor of 10 to aid interpretation: An increase in EQ-5D index of 0.1 equates to a 0.845 times change in odds of achieving either OHS PASS or MIC

^+^ASA grade II as reference category

## Discussion

This study demonstrated that the PASS for the OHS following rTHA was 31.5 at one year, which increased to 33.5 at two years postoperatively. The MIC was 8.5 at both one and two year follow-up. At both time points, greater preoperative HRQoL was independently associated with PASS achievement, and lower preoperative OHS was associated with MIC achievement. Lower preoperative HRQoL and poorer physical health (ASA) were associated with failure to achieve either satisfactory state at one or two years postoperatively.

Whilst this is the first study, to the authors’ knowledge, to define the OHS PASS following rTHA, the threshold scores are at the lower range of those reported following primary THA, which vary from 30.6 to 40 points [7, 19–21]. This range of values reflects that PROM CMVs may be dependent on a variety of factors that are often specific to the population assessed [22]. Outcomes following primary THA are more predictable than rTHA even in the absence of infection, and greater postoperative functional outcomes have been reported for primary THA than rTHA in comparative cohorts [2]. This is reflected by the fact that PASS thresholds following rTHA appear lower than those after primary THA, which may suggest that patient expectations regarding their postoperative function are slightly lessened in the event of revision surgery.

The OHS MIC thresholds of 8.5 at one and two years postoperatively in the reported cohort following rTHA are 2.5 points greater than the MIC of 6 reported by Sabah et al*.* at six months postoperatively [8]. Whilst this difference is less than the described measurement error of the OHS [23], small increases in postoperative OHS have been reported between six and 12-months postoperatively following rTHA, which may be reflected in patient expectations for a satisfactory improvement [24]. MIC thresholds following primary THA are less than the MIC reported here following rTHA, with Harris et al*.* reporting MIC values of 6.1 and 6.0 at one and two years postoperatively, respectively [9]. Whilst comparisons between primary and rTHA CMVs are likely limited by the differing populations and expectations, more severe preoperative symptomatology is associated with greater OHS MIC values [19]. Furthermore, this plateau in OHS PASS and MIC thresholds from one and two years postoperatively after primary THA mirrors the plateau reported in absolute OHS score beyond one year postoperatively [7, 10, 19]. Similarly, the PASS and MIC thresholds described in this study for rTHA remain largely unchanged over the same timeframe.

This study identified that greater preoperative HRQoL (EQ-5D) was independently associated with both one and two year PASS achievement. Similarly a range of factors, including anxiety and depression, pain catastrophising, preoperative function, and preoperative HRQoL, have been associated with PASS achievement in hip-specific PROMs, albeit after primary THA [25–27]. The preoperative EQ-5D thresholds of 0.165 and 0.178 showed excellent capacity to discriminate which patients were likely to achieve PASS at one and two years postoperatively, respectively. Conversely, MIC achievement was associated with lower preoperative OHS at both one and two years postoperatively. Whilst not described for the MIC, this has been observed for the minimum clinically important difference (MCID) in primary THA [28, 29]. Greater preoperative function is associated with greater postoperative function, and this inverse association with MIC achievement likely reflects that patients with worse preoperative OHS have greater potential for improvement, less constrained by ceiling effects. This highlights the necessity of considering both PASS and MIC thresholds in conjunction when assessing outcomes, as failure to achieve one in isolation does not necessarily equate to a poor outcome for the patient. Reduced BMI was also independently associated with PASS achievement at two years postoperatively, with obese patients’ odds of MIC achievement just 28.5% compared to non-obese when adjusting for preoperative function. Obesity has similarly been shown to be associated with MCID achievement after primary THA, but not with MIC achievement, nor after revision arthroplasty [29]. However, increased BMI is associated with increased complication risk and poorer functional outcomes following rTHA, which may explain this finding [30, 31].

Preoperative HRQoL (EQ-5D) and preoperative health state (ASA grade) were strongly associated with failure to achieve either the PASS and or the MIC for the OHS following rTHA at one and two years postoperatively, respectively. The failure to achieve either CMV was associated with dissatisfaction in 75% of cases at one year; greater than the 42.9% associated with failure to achieve MIC in isolation, and the 56.2% associated with failure to achieve PASS. Whilst dissatisfaction after rTHA is multifactorial and underpinned by patient expectations [32], increasing ASA grade is associated with increased risk of poor functional outcomes, which may explain the association between ASA and failure to achieve either CMV at two years postoperatively [29]. Failure to achieve either CMV was uncommon, with the group consisting of just eight cases by two years postoperatively. This necessitates caution when interpreting model findings, which should be viewed as exploratory.

The findings from this study are limited by the attrition to PROM completion, with 66.7% completion preoperatively, falling to 59.4% at one year and 51.4% at two years postoperatively. However, 73.9% of patients completed either postoperative follow-up, so the consistent findings observed between one to two year CMV thresholds and associated models are reassuring. The small sample size in this cohort necessitated parsimonious model building and introduces the risk of type 2 error. The follow-up duration is appropriate to define early CMV thresholds but is likely inadequate to assess survivorship. A further limitation when assessing rTHA is the heterogeneity in the surgical procedures performed, with patients receiving bone grafting, augmentation, and dual mobility constructs on a case-by-case basis. However, all rTHA performed in this prospective cohort had a standardised surgical technique using the same acetabular component, and all patients underwent rTHA for aseptic indications, mitigating against the established differences in outcomes between this and rTHA for infection [12].

## Conclusions

The PASS and MIC thresholds for the OHS following aseptic rTHA contextualise the score and can inform study design. Greater preoperative HRQoL (EQ-5D) was independently associated with PASS achievement, whilst worse preoperative function was independently associated with MIC achievement. These thresholds should be considered in conjunction when assessing outcomes following aseptic rTHA.

## Data Availability

No datasets were generated or analysed during the current study.
